# Roll-Out Deployment Process Analysis of a Fiber Reinforced Polymer (FRP) Composite Tape-Spring Boom

**DOI:** 10.3390/polym15112455

**Published:** 2023-05-25

**Authors:** Sicong Wang, Shuhong Xu, Lei Lu, Lining Sun

**Affiliations:** 1 School of Mechanical and Electrical Engineering, Soochow University, Suzhou 215137, China; scwang8901@suda.edu.cn (S.W.);; 2 School of Engineering, Applied Technology Collage of Soochow University, Suzhou 215325, China

**Keywords:** FRP, deployable boom, tape-spring, roll-out, bistable

## Abstract

Deployable extendable booms are widely used in aerospace technology due to many advantages they have, such as high folded-ratio, lightweight and self-deployable properties. A bistable FRP composite boom can not only extend its tip outwards with a corresponding rotation speed on the hub, but can also drive the hub rolling outwards with a fixed boom tip, which is commonly called roll-out deployment. In a bistable boom’s roll-out deployment process, the second stability can keep the coiled section from chaos without introducing a controlling mechanism. Because of this, the boom’s roll-out deployment velocity is not under control, and a high moving speed at the end will give the structure a big impact. Therefore, predicting the velocity in this whole deployment process is necessary to be researched. This paper aims to analyze the roll-out deployment process of a bistable FRP composite tape-spring boom. First, based on the Classical Laminate Theory, a dynamic analytical model of a bistable boom is established through the energy method. Afterwards, an experiment is introduced to produce some practical verification for comparison with the analytical results. According to the comparison with the experiment, the analytical model is verified for predicting the deployment velocity when the boom is relatively short, which can cover most booms using CubeSats. Finally, a parametric study reveals the relationship between the boom properties and the deployment behaviors. The research of this paper will give some guidance to the design of a composite roll-out deployable boom.

## 1. Introduction and Literature Review

**Figure 1:**
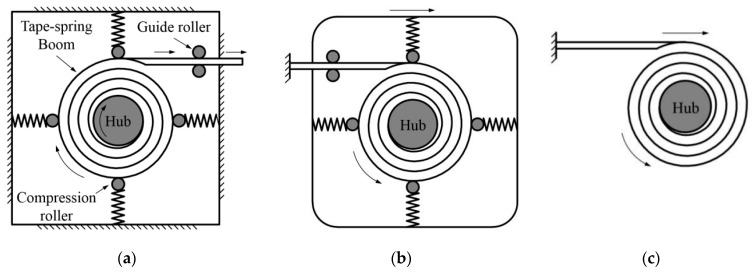
Tape-spring boom deployment modes. (**a**) Normal deployment (**b**) Roll-out deployment (with rollers) (**c**) Roll-out deployment (no rollers necessary).

Extendable tubular booms, such as a carpenter’s tape (see Figure 1), are widely used on spacecraft structures such as solar sails, antennas and solar arrays. This should thank to their high folded-ratio, simple deployment mechanism, low mass properties, etc. [1]. The first version of these booms, which was called Storable Tubular Extendable Member (STEM), was invented by Klein, using a satellite antenna [2]. Although the early study of the booms was mainly concentrated on isotropic ones, which were usually manufactured by metals, for instance, steel or CuBe, composite materials with laminated layers were commonly used in recent years as the laminated booms could be designed flexibly according to the requirements in applications [1]. A typical deployment mode of the booms is shown in Figure 1a where the tip extends outwards continuously with a corresponding rotation speed on the hub, which is connected by an actuating motor, and the rollers around the controlling mechanism are pressing on the coiled section of the boom to keep the boom from chaos. Since a mono-stable boom was likely to be chaotic in the deployment process, a controlling mechanism was necessary to be introduced during the deployment. Therefore, bistable booms, which were made by certain laminate layout, were invented to improve the boom’s deployment reliability and to reduce the total mass of the whole structure, as a bistable boom could be not only stable at its wholly deployed state (first stability) but also keep stable at its fully folded state (second stability) [3,4]. More importantly, a bistable boom could either extend its tip outwards with a fixed but rotatable hub (as shown in Figure 1a) or roll and push the hub moving outwards with a tip fully fixed (which was commonly called roll-out deployment mode, see Figure 1b). In the boom’s roll-out deployment process, the constrained mechanism was not necessarily to be used any more (shown in Figure 1c) because the second stability of the boom could already keep the coiled section stable before deployment and hence make the deployment process sequential [5]. However, just because of this, the movement velocity of the hub could not be controlled by a motor as no controlling mechanism was introduced in the roll-out deployment mode. Meanwhile, the velocity, especially at the end of the deployment, was necessary to be predicted as an extra fast hub movement would break the structures the boom connected, for example, the membranes, and influence the in-orbit attitude of the satellite. In the previous work, Mallol conducted some dynamic analysis of bistable booms putting into a controlling mechanism, and some simulations and experiments were carried out to afford some practical verification [6]. Meanwhile, Tibert from KTH analyzed the impact of the viscoelasticity (stemming from the boom’s stowage time) on the deployment duration of a boom, and the damping factors of the laminate materials were finally acquired [7]. Pellegrion from Caltech investigated a bistable cylindrical shell, and a comprehensive analytical model was developed which could predict the residual stress distribution and bistable configurations of the shell [8,9,10]. Further, Pellegrino also concentrated on the analysis of multi-stable morphing structures, for instance, self-deployable shells, in the recent work for extending the applications of the FRP composite materials [11,12,13].

Otherwise, for the abnormal deployment types of the tape-spring booms, blossoming is also a failure mode during the boom’s deployment process. For the sake of analyzing and predicting the critical conditions of blossoming, Iqbal and Pellegrino established an analytical model of a metal boom based on the strain energy principle [14]. The model was upgraded by Wang by introducing a finite element model of the mechanisms, and then the pressure distribution of the boom’s coiled section caused by the compression rollers on the constraint mechanisms was finally acquired [15]. Wang also analyzed the blossoming behaviors of an FRP composite tape-spring boom, and the maximum tip load a boom could afford before blossoming happened was found eventually [16]. Furthermore, with respect to the analysis of the FRP composites, Koloor produced an energy-based concept for multi-directional composite structures. Based on the damage dissipation energy, the yield point of the material could be found according to the new method proposed [17]. Deifalla established a machine-learning model for FRP concrete beams. This model was able to calculate the ultimate torsion strength of the composite with externally bonded FRP materials [18]. Moreover, a new approach method for flexural strength prediction was proposed by Amin, which was used to predict the moment bearing capacity of the beam under bending loads [19]. Admin also built a tree-based prediction model for externally bonded FRP laminates based on varying genetic parameters [20]. Moreover, in recent research, Liu proposed a new kind of 2D and 3D chiral mechanical metamaterials based on prestressed bistable metallic shells to resolve the issue that morphing strategies are rare [21].

From the literature review above, the former research did not focus on analyzing the deployment velocity during the full deployment process of the boom to acquire the relationships between the boom’s deployed length and deploying velocity. In contrast, an excessively high velocity could impact the on-orbit attitude of the satellite and even play a devil with the whole mission. Therefore, this paper concentrates on predicting the deployment velocity of a bistable Storable Tubular Extendable Member (STEM) boom in its roll-out deployment process. Note that the booms with the other typical cross-sections, such as Collapsible Tubular Member (CTM) booms and Triangular Rollable And Collapsible (TRAC) booms, were also suitable for the analytical model established in this paper. In contrast, a slit-tube STEM boom was selected as a representative to make the illustration. Section 2 analyzes the deployment process of a bistable composite STEM boom, and the deployment velocity during the whole process could be acquired by establishing an analytical model using the energy method based on the Classical Laminate Theory. Furthermore, in Section 3, an experiment is introduced to provide some practical verification for the analytical model built in the second section. Section 4 explores the relationships between the roll-out deployment behaviors and the boom geometric and material parameters and provides some guidance for the parametric design of a hybrid roll-out deployment FRP tape-spring boom was obtained. Finally, Section 5 concludes the paper and has a discussion.

## 2. Deployment Process Dynamic Analysis of a Bistable STEM Boom

Since the interactions of the boom infinitesimals during the deployment are quite complicated, a strain energy model is considered to be used to describe the deployment behaviors.

### 2.1. Strain Energy Model Establishment

**Figure 2:**
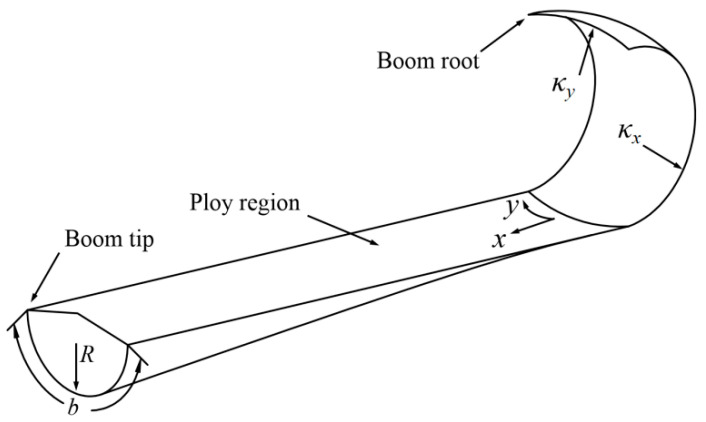
STEM boom parameter diagram.

As the thickness of the boom is much smaller than its natural radius and coiling radius, the boom can be assumed as a thin-walled structure. The Classical Laminate Theory (CLT) is introduced to calculate the boom’s elastic properties, which can be expressed by an ABD matrix as [22]:

(1) $$\left[\begin{array}{c} N_{x}\\ N_{y}\\ N_{xy}\\ M_{x}\\ M_{y}\\ M_{xy} \end{array}\right]=\left[\begin{array}{cccccc} A_{11} & A_{12} & A_{16} & B_{11} & B_{12} & B_{16}\\ A_{12} & A_{22} & A_{26} & B_{12} & B_{22} & B_{26}\\ A_{16} & A_{26} & A_{66} & B_{16} & B_{26} & B_{66}\\ B_{11} & B_{12} & B_{16} & D_{11} & D_{12} & D_{16}\\ B_{12} & B_{22} & B_{26} & D_{12} & D_{22} & D_{26}\\ B_{16} & B_{26} & B_{66} & D_{16} & D_{26} & D_{66} \end{array}\right]\left[\begin{array}{c} \varepsilon_{x}\\ \varepsilon_{y}\\ \gamma_{xy}\\ \kappa_{x}\\ \kappa_{y}\\ \kappa_{xy} \end{array}\right]$$

which can be expressed compactly as:

(2) $$\left[\begin{array}{c} N\\ M \end{array}\right]=\left[\begin{array}{cc} A & B\\ B & D \end{array}\right]\left[\begin{array}{c} \varepsilon \\ \kappa \end{array}\right]$$

where *N_x_*, *N_y_*, and *N_xy_* are the stretching force per unit length, *M_x_*, *M_y_*, and *M_xy_* are the bending torque per unit length, *ε_x_*, *ε_y_*, and *γ_xy_* are stretching and shearing strain and *κ_x_*, *κ_y_*, and *κ_xy_* are bending and twisting curvatures. Note that *κ_x_* is the curvature along the boom’s longitudinal cross-section, and *κ_y_* is the transversal cross-section curvature (parameters above shown in Figure 2).

Since bistable booms manufactured for roll-out deployment mode are commonly laminated symmetrically, the matrix ***B*** in Equation (2) should be zero [9]. Furthermore, *D*_16_ and *D*_26_ can also be assumed to be zero as the coupling between the boom’s bending and twisting behaviors has less effect on the boom deployment behaviors. Furthermore, *κ_xy_* would be ignored as well because the behavior caused by *κ_xy_* cannot be revealed when the boom is working ideally. Meanwhile, with regard to matrix ***A***, only the stretching behavior along the boom longitudinal direction (*A*_11_) is obvious [16]. Therefore, according to the analysis above, Equation (1) can be simplified as follows:

(3) $$\left[\begin{array}{c} M_{x}\\ M_{y} \end{array}\right]=\left[\begin{array}{cc} D_{11} & D_{12}\\ D_{12} & D_{22} \end{array}\right]\left[\begin{array}{c} \kappa_{x}\\ \kappa_{y} \end{array}\right]$$

and

(4) $$N_{x}=A_{11}\varepsilon_{x}$$

which presents the boom’s bending and stretching elastic behaviors during the deployment process, respectively.

According to Ref. [23], the bending and stretching energy of the boom coiled section (i.e., the energy of the whole boom as the strain energy of the boom transition zone (also called *ploy region* in Figure 2) can be ignored since the energy in this section always keeps constant during the deployment process) would be calculated through the equations as follows:

(5) $$\begin{array}{l} U_{B}=\frac{b}{2}\left[\begin{array}{ccc} \kappa_{x} & \kappa_{y} & \kappa_{xy} \end{array}\right]D\left[\begin{array}{c} \kappa_{x}\\ \kappa_{y}\\ \kappa_{xy} \end{array}\right]\\ =\frac{b}{2}\left(D_{11}\Delta \kappa_{x}^{2}-2D_{12}\Delta \kappa_{x}\Delta \kappa_{y}+D_{22}\Delta \kappa_{y}^{2}\right) \end{array}$$

and

(6) $$U_{S}=\frac{b}{2}\left[\begin{array}{ccc} \varepsilon_{x} & \varepsilon_{y} & \varepsilon_{xy} \end{array}\right]A\left[\begin{array}{c} \varepsilon_{x}\\ \varepsilon_{y}\\ \varepsilon_{xy} \end{array}\right]=\frac{b}{2}A_{11}\varepsilon_{x}^{2}$$

where *b* is the path length along the boom transversal cross-section (which can be seen in Figure 2).

Based on Equations (1)–(6), the boom’s strain energy per unit length *e* and the total energy of the boom in the deployment process can be expressed respectively by [14]:

(7) $$\begin{array}{l} e=\frac{b}{2}\left[D_{11}\kappa_{x}^{2}+2D_{12}\kappa_{x}\left(\kappa_{y}-\frac{1}{R}\right)+D_{22}{\left(\kappa_{y}-\frac{1}{R}\right)}^{2}\right]\\ +\frac{1}{\kappa_{y}}\frac{A_{11}}{2}\left[\frac{b}{2}\frac{\kappa_{x}^{2}}{\kappa_{y}^{2}}-\frac{4\sin^{2}\left(\frac{b\kappa_{y}}{2}\right)}{b}\frac{\kappa_{x}^{2}}{\kappa_{y}^{3}}\right] \end{array}$$

(8) $$E\left(\alpha_{1}\right)=\int_{0}^{\alpha_{b}-\alpha_{1}}\left(r_{i}+a\alpha \right)e_{\min}\left(\alpha \right)d\alpha$$

where *b* is the path length along the boom cross-section, *R* is the initial/natural radius of the cross-section (see Figure 2), *α*_1_ is the hub rotation angle from the initial state during the deployment, *r_i_* is the coiled radius of the boom root, *e*_min_ is the minimum strain energy per unit length acquired by the minimum energy principle which is illustrated in Ref. [14] in details, and *α_b_* is the hub rotation angle deploying from the start to the end which can be acquired by:

(9) $$l_{b}=\int_{0}^{\alpha_{b}}r\left(\alpha \right)d\alpha =\int_{0}^{\alpha_{b}}\left(r_{h}+\frac{T}{2}+\frac{\alpha T}{2\pi }\right)d\alpha$$

where *l_b_* is the total length of the boom, *r_h_* is the hub radius, and *T* is the boom’s wall thickness.

Note that the equations in this paper are used to predict the deployment behavior of the booms whose hubs are on the same sides as the booms’ concave surfaces (commonly called *equal-sense coiling*). However, for the *opposite-sense coiling* booms, this method can also be used only by changing the positive sign in front of the second term to negative in Equation (5).

### 2.2. Boom Deployment Process Analysis

Based on the strain energy model built in Section 2.1, from Equation (8), the boom roll-out deployment/driving force *F* would be acquired by

(10) $$F\left(\alpha_{1}\right)=\frac{dE\left(\alpha_{1}\right)}{d\alpha_{1}}r\left(\alpha_{1}\right)$$

In the boom deployment process, some practical effects, such as fraction between the ploy region and coiled section, viscoelasticity in the fiber resin and air damping, can slow down this movement. For the sake of illustration, the damping factor *μ* is introduced to describe the factor. It can be assumed that factor *μ* keeps constant during the deployment when the kinematic velocity of the hub is relatively low (i.e., the boom is not excessively long).

**Figure 3:**
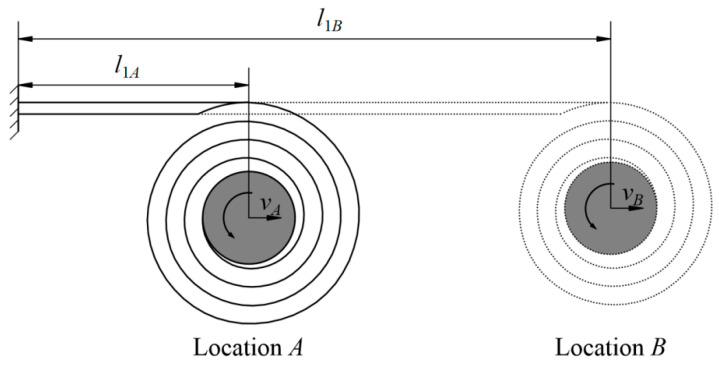
Parameters in boom deployment process.

After introducing the damping factor *μ*, the kinetic equation of the boom deployment process is expressed as:

(11) $$\begin{array}{l} \int_{0}^{l_{1}}F\left(l\right)\left(1-\mu \right)dl\\ =\frac{1}{2}\left(m_{s}+m_{b}\right)v_{1}^{2}+\frac{1}{2}\left(J_{s}+J_{b}\right){\left[\frac{v_{1}}{r\left(\alpha_{1}\right)}\right]}^{2} \end{array}$$

where *l* is the variable of integration, *l*_1_ is the deployed length (corresponding with *α*_1_, 0 ≤ *l*_1_ ≤ *l_b_*, see Figure 3), *m_s_* and *m_b_* are the mass of the hub and the boom coiled/undeployed section, *J_s_* and *J_b_* are the rotational inertia of the hub and the boom coiled section, and *v*_1_ is the hub movement velocity with a deployed length *l*_1_. The terms *m_b_*, *J_s_* and *J_b_* in Equation (11) can be found by:

(12) $$m_{b}=\rho_{b}\left(l_{b}-l_{1}\right)$$

(13) $$J_{s}=\frac{m_{s}\left[R^{2}-{\left(R-t_{h}\right)}^{2}\right]}{2}$$

(14) $$J_{b}=\frac{\rho_{b}}{2}\int_{0}^{\alpha_{b}-\alpha_{1}}r\left(\alpha \right)\left\{r^{2}\left(\alpha \right)+{\left[r\left(\alpha \right)-t\right]}^{2}\right\}d\alpha$$

in which *ρ_b_* is the boom linear density, and *t_h_* is the hub wall thickness.

Combining Equations (10) and (12)–(14) into Equation (11), the deployment velocity of the hub center under different deployed lengths/locations (as shown in Figure 3) can be obtained through

(15) $$v_{1}\left(\alpha_{1}\right)=\sqrt{\frac{2\int_{0}^{l_{1}\left(\alpha_{1}\right)}F\left(l\right)\left(1-\mu \right)dl}{m_{s}+m_{b}+\frac{J_{s}+J_{b}}{r^{2}\left(\alpha_{1}\right)}}}$$

where *α*_1_ is the only independent value in this equation.

## 3. Deployment Analysis and Experimental Comparison

For verifying the analytical model established in Section 2, an experiment was introduced to make a comparison. Two boom samples were used in the experiments for illustration, and the samples in the experiment were both manufactured by Carbon Fiber Reinforced Polymers (CFRP), which were laminated in three layers: one unidirectional (UD) layer with 0° angle in the middle of two fabric layers with ±*θ* fiber angles on both sides (±*θ* was regarded as one laminate layer in this paper). As is commonly used on many CFRP booms, the fiber angles of the two fabric layers were selected the same, and the layout of the boom laminate was set symmetrically. The booms’ geometric and material parameters used in the experiment, which are shown in Table 1 and Table 2, respectively, were used to mimic those used in the InflateSail CubeSat [24], in which *E_m_*, *G_m_*, and *v_m_* are the elastic modulus, shear modulus, Poisson’s ratio of the matrix, *E_f_*, *G_f_*, and *v_f_* are the elastic modulus, shear modulus, Poisson’s ratio of the fiber, *T*_UD_, *V*_UD_, and *Φ*_UD_ are the thickness, volume fraction, porosity of the unidirectional (UD) ply and the *T_f_*, *V_f_*, and *Φ_f_* are the thickness, volume fraction, and porosity of each fabric ply. Note that the introducing method of the material parameters in Table 2 into the equations in Section 2 was commonly used in the mechanics of composite laminate materials, which could also be found in Ref. [1]. No hub was used in the experiment (i.e., *m_s_* = *J_s_* = 0 in Equations (11) and (13)). The two boom samples in the experiment were manufactured with the same geometric and material parameters listed in Table 1 and Table 2, except the laminate layout was assigned as [±45°F/0°/±45°F] (Sample 1) and [±50°F/0°/±50°F] (Sample 2), respectively.

**Figure 4:**
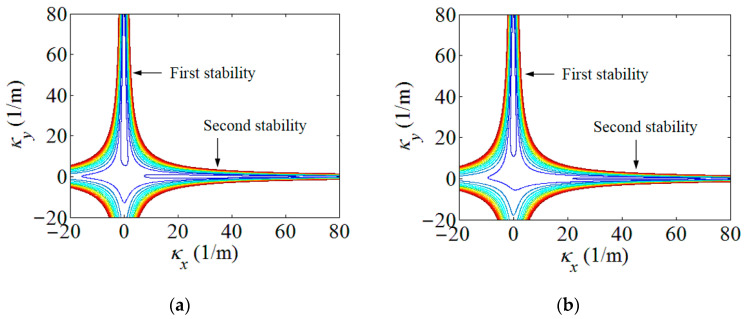
Boom infinitesimal strain energy contour. (**a**) Boom Sample 1; (**b**) Boom Sample 2.

**Figure 5:**
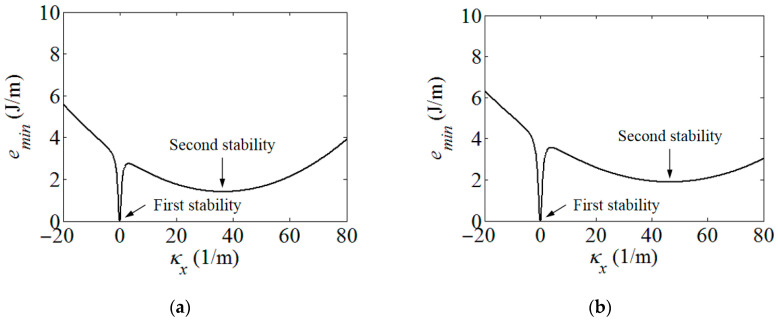
Boom strain energy per unit length under different coiled radii. (**a**) Boom Sample 1; (**b**) Boom Sample 2.

**Figure 6:**
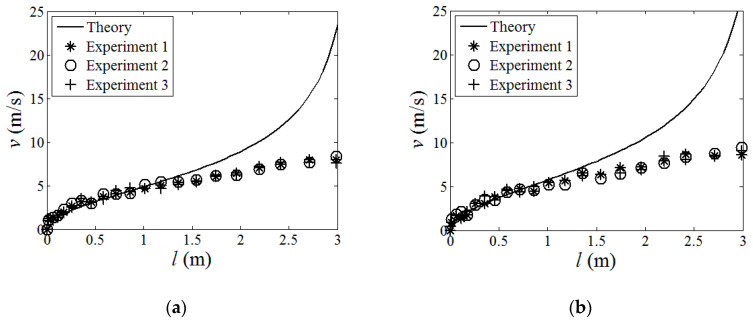
Analytical and experimental results comparison. (**a**) Boom Sample 1; (**b**) Boom Sample 2.

**Figure 7:**
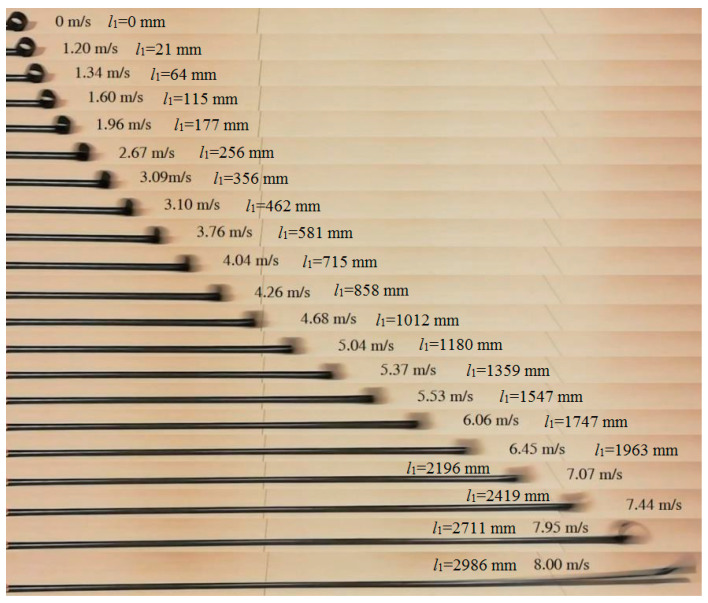
Boom deployment process in the experiment (Sample 1 as a representative).

Using the strain energy model built in Section 2, the energy contours for the infinitesimals of the boom samples used in the experiment could be acquired in Figure 4. For presenting the linear strain energy density along the boom length, the strain energy per unit length under different bending curvatures, i.e., different *κ_x_*, were further plotted in Figure 5. Note that the plots in Figure 5 were the energy integrals along the boom cross-sections based on the data in Figure 4 for better viewing. From Figure 4 and Figure 5, it could be found that each boom sample had two minimum energy value points. That was to say that each boom had two energy stabilities, which was a bistable tape-spring boom. One of the stabilities was at a boom’s fully deployed (initial) state (*κ_x_*_1_ = *κ_x_*_2_ = 0 and *κ_y_*_1_ = *κ_y_*_2_ = 1/*R* = 50 m^−1^), which was called the first stability in this paper for the sake of illustration. Additionally, the curvature of the other/second stabilities of the two samples were *κ_x_*_1_ = 36.4 m^−1^ and *κ_x_*_2_ = 46.4 m^−1^, i.e., the curvature radii were *r_x_*_1_ = 27.5 mm and *r_x_*_2_ = 21.6 mm, respectively, according to the plots in Figure 5. The areas apart from the stable points in Figure 4 and Figure 5 were unstable regions which were the transition stages (boom deforming process) during the deployment. Since there was no hub introduced in this experiment, the curvature radii of the booms’ second stabilities were regarded as the hub radii, i.e., *r_h_*_1_ = *r_x_*_1_ = 27.5 mm and *r_h_*_2_ = *r_x_*_2_ = 21.6 mm. According to the experimental experience presented in Ref. [23], for a three-layer fabric laminate boom, the damping factor would be selected as *μ* = 0.72. By introducing the damping factor *μ* into the theoretical model in Section 2, the comparison of the experimental and the analytical results can be seen in Figure 6, and the deployment process of boom Sample 1 in the experiment is shown in Figure 7 as a representative, in which the instantaneous velocities and deployed lengths were marked and the time increment of each frame was 1/30 s for illustration. Three repeated tests were carried out for each boom sample in the experiment in order to improve reliability.

From the experiment, through comparing the results listed in Figure 6, it could be found that the experimental and theoretical results matched quite well with each other at the first half of the deployment process both for Sample 1 and Sample 2 when the deployment velocities were relatively low, i.e., the deployed length was relatively short. Meanwhile, the results from the three tests for each boom sample were consistent. Therefore, from the experiment, it could be known that the boom’s deployment experiment was credible, and the analytical model established in Section 2 was available for describing the deployment behaviors of a roll-out deployment boom when the whole length of the boom was not too long. Moreover, in the first half of the deployment, the velocities of the samples increased rapidly at the beginning, yet, as the deployment proceeded, the accelerations of the booms slightly decreased. These cases could be found both from the theoretical and experimental results since the increasing velocities could lead to the increase of the damping forces (not the damping factor) in Equation (11).

However, in the second half of the process of Sample 1 and Sample 2, the practical results were gradually lower than the analytical theory. In this stage, the theory continued going up while the velocities of the samples were approaching constant values. This was because, as the moving velocities increased, the damping factor *μ* in Equation (15) should not be regarded as a constant value anymore. That is to say, the prediction of the factor *μ* needed to be modified and upgraded to more accurate values for high-speed boom deployment, and, for a boom with a longer deployed length, a varying factor function of the deployment velocity *μ*(*v*_1_) should be acquired, and this will be investigated in the future work. Nevertheless, the analytical model in Section 2 with a constant *μ* could still be used to predict the deployment process of a relatively short tape-spring boom (less than 1.5 m according to the experiment, which could cover most booms using CubeSats). For example, in Figure 6, the samples’ velocities were nearly constant near the end of the deployment as the damping forces were approaching the booms’ driving forces during this section.

To sum up, the experimental results were consistent, and the analytical method in Section II was credible when the deployment length was relatively short (most booms used for CubeSats could be covered). However, when the boom was longer, a more accurate damping factor *μ*(*v*_1_) which was a function of the deployment velocity, was needed for further investigation.

## 4. Parametric Study

**Figure 8:**
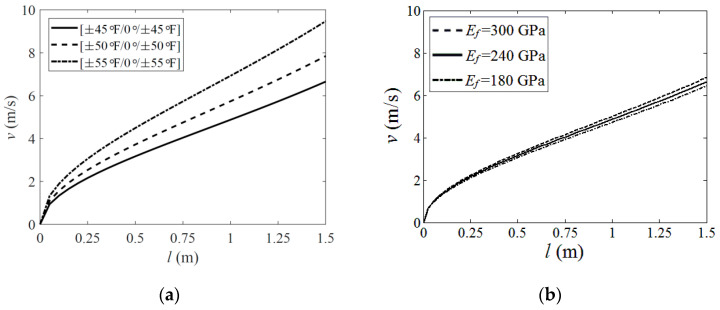
Material parametric analysis. (**a**) Fiber angle; (**b**) Fiber stiffness.

**Figure 9:**
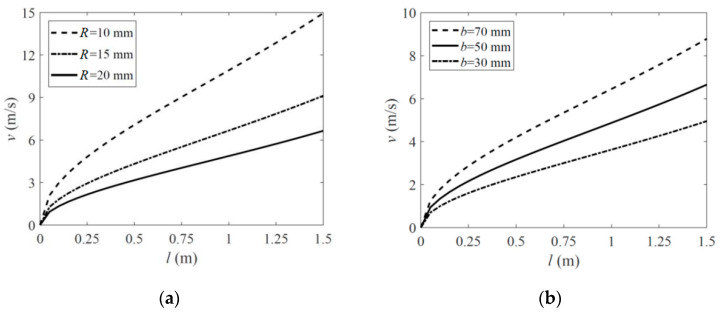
Geometric parametric analysis. (**a**) Natural radius; (**b**) Path length.

Based on the analysis in Section 2 and Section 3, a parametric study was carried out to explore the influence of the boom’s geometric and material properties on its deployment velocity when the wholly deployed length of the boom was relatively short (up to 1.5 m in this investigation). Several typical parameters were selected as follows: the ply angles of the fabric layers and the fiber stiffness *E_f_* whose influences were given in Figure 8, and the boom’s natural cross-section radius *R* and path length *b,* whose effects were listed in Figure 9. The parameters which were not marked in Figure 8 and Figure 9 were the same as those listed in Table 1 and Table 2 (fabric plies laminate layout for the plots in Figure 8b and Figure 9a,b was all set as [±45°F/0°/±45°F]).

According to the results in Figure 8a, the increment of the deployment velocity grew with an increasing fiber angle. From Figure 8b, it could also be found that higher fiber stiffness led to higher deployment velocity, while the change was relatively insensitive. This was because, for a boom with a higher ply angle or higher fiber stiffness, more energy was needed to be input into the layers when the boom was flattened before the deployment. Although higher ply angles could also make the boom acquire less energy when bent or coiled on the hub along its longitudinal direction, this reduction still could not turn the scale. A similar mechanism appeared when the natural radius *R* was changing, as shown in Figure 9a. A boom with a smaller natural radius (while the path length *b* was constant) acquired more strain energy when flattened and thus could produce a higher driving force in the deployment process. Meanwhile, the velocity results in Figure 9a presented nonlinear variation with the change of *R*, and the variation was relatively sensitive. Otherwise, the velocity increment increased linearly with a growing path length *b*, and this could also be known by Equation (2) in Section 2.

According to the analysis above, the deployment velocity could be limited by reducing the laminate angle. However, in the meantime, a lower ply angle boom required a hub with a higher radius *r_h_* because a boom with lower ply angles would have a larger natural coiled radius *r_n,_* and the hub radius should be smaller than the boom’s natural coiled radius to keep the boom coiling on the hub tightly (see Ref. [16] for more details), and this would pump up the folded volume of the mechanism. Further, a smaller natural radius *R* or shorter path length *b* could lead to a lower driving force as well, and, meanwhile, this reduction was also able to decrease the boom’s bending stiffness when wholly deployed. Therefore, from the analysis above, the parametric design of the roll-out deployable boom should consider the deployment velocity, the folded volume and the deployed stiffness comprehensively.

## 5. Conclusions and Discussion

A bistable tape-spring boom can deploy in the form of roll-out mode in sequence, even if there is no controlling mechanism introduced to keep the boom from chaos. Just because of this, no motor is used to control the boom’s deployment velocity, and an excessively high velocity at the end would rush and damage the boom and the other structures connected, for instance, the membrane. Therefore, predicting the velocity in the boom’s roll-out deployment process is necessary to be researched.

Based on the CLT, this paper established an analytical model from the aspect of energy method to describe the boom roll-out deployment process, and the hub velocity at each location of the deployment was acquired. Afterwards, an experimental study was presented to provide some practical verification. According to the experiment, the practical results were consistent, and the theoretical velocity prediction was accurate when the boom length was relatively short (up to 1.5 m, which could cover most FRP booms used for CubeSats). However, when the boom was much longer, the experimental results would be gradually lower than those acquired from the theory at the latter half of the deployment because the damping factor in the analytical model was not linearly dependent on the driving force anymore when the deployment velocity was relatively high. Meanwhile, the practical velocity increased more gently in the latter stages of the deployment and was even nearly constant when approaching the end since the damping force was approaching the boom’s driving force in this case, while the theory was still going up more rapidly. To describe these cases precisely, the damping factor needed to be substituted by a function of the boom’s deployment velocity, and this point will be further researched in future investigations.

Moreover, a parametric study was carried out to explore the effect of the boom’s geometric and material parameters on the deployment process. According to the analysis, the deployment velocity increased when the boom had a larger fiber angle, a higher fiber stiffness (less sensitive), a smaller natural radius or a longer cross-section path length because these changes would increase the amount of the strain energy required when the boom was flattened before the deployment, and vise versa. However, although the changes could also reduce the boom’s longitudinal energy input when coiling on the hub, this factor was not strong enough to turn the scale. In the meantime, the changes in the parameters for reducing the driving forces or the tip speed could also influence the stiffness properties of the boom when fully deployed or the folded volume when coiled. Therefore, the parametric design of a tape-spring boom needed to be considered comprehensively.

The theoretical model established and the analysis carried out in this paper will provide some guidance in the design of a bistable roll-out self-deployment tape-spring FRP boom structure mechanism.

## Figures and Tables

**Table 1:** Tip-spring boom sample geometric parameters.

*R* (mm)	*b* (mm)	*l_b_* (m)
20	50	3

**Table 2:** Tip-spring boom sample laminate parameters.

*E_m_* (GPa)	*G_m_* (GPa)	*v_m_*	*E_f_* (GPa)
4	2.7	0.35	240
*G_f_* (GPa)	*v_f_*	*T*_UD_ (mm)	*V*_UD_ (%)
95	0.22	0.057	31
*Φ*_UD_ (%)	*T_f_* (mm)	*V_f_* (%)	*Φ_f_* (%)
85	0.096	53	85

## Data Availability

The data presented in this study are available on request from the corresponding author. The data are not publicly available due to relevant policy restrictions.

## References

[1] Daniel I.M., Ishai O. (1994). Engineering Mechanics of Composite Materials.

[2] Bourgeois-Doyle R.I., George J.K. (2004). The Great Inventor.

[3] Daton-Lovett A. (2001). Extendible Member. U.S. Patent.

[4] Mao H., Tibert G. Experiment and Analytical Modeling for Designing tape-spring Composites. Proceedings of the 21st International Conference on Composite Materials.

[5] Hoang B., White S., Spence B., Kiefer S. Commercialization of Deployable Space Systems’ Roll-out Solar Array (ROSA) Technology for Space Systems Loral (SSL) Solar Arrays. Proceedings of the 2016 IEEE Aerospace Conference.

[6] Mallol P., Tibert G. Deployment Modeling and Experimental Testing of a Bi-stable Composite Boom for Small Satellites. Proceedings of the AIAA/ASME/ASCE/AHS/ASC Structures, Structural Dynamics, & Materials Conference.

[7] Mao H., Shipsha A., Tibert G. (2017). Desigh and Analysis of Laminates for Self-Deployment of Viscoelastic Bistable tape-springs After Long-term Stowage. J. Appl. Mech..

[8] Kebadze E., Guest D., Pellegrino S. (2004). Bistable Prestressed Shell Structures. Int. J. Solids Struct..

[9] Iqbal K., Pellegrino S., Daton-Lovett A. (2004). Bistable Composite Slit Tubes. IUTAM-IASS Symposium on Deployable Structures: Theory and Applications.

[10] Wilson L., Gdoutos E.E., Pellegrino S. (2019). Tension-Stabilized Coiling of Isotropic Tape Springs. Int. J. Solids Struct..

[11] Leclerc C., Pellegrino S. (2020). Nonlinear Elastic Buckling of Ultra-thin Coilable Booms. Int. J. Solids Struct..

[12] Li Y., Pellegrino S. (2020). A Theory for the Design of Multi-stable Morphing Structures. J. Mech. Phys. Solids.

[13] Ferraro S., Pellegrino S. (2021). Topology and Shape Optimization of Ultrathin Composite Self-Deployable Shell Structures with Cutouts. AIAA J..

[14] Guest S.D., Pellegrino S. (2006). Analytical models for bistable cylindrical shells. Proc. R. Soc. A Math. Phys. Eng. Sci..

[15] Wang S., Schenk M., Guo H., Viquerat A. (2020). Tip Force and Pressure Distribution Analysis of a Deployable Boom During Blossoming. Int. J. Solids Struct..

[16] Wang S., Schenk M., Jiang S., Viquerat A. (2020). Blossoming Analysis of Composite Deployable Booms. Thin-Walled Struct..

[17] Rahimian Koloor S.S., Karimzadeh A., Yidris N., Petrů M., Ayatollahi M.R., Tamin M.N. (2020). An Energy-Based Concept for Yielding of Multidirectional FRP Composite Structures Using a Mesoscale Lamina Damage Model. Polymers.

[18] Deifalla A., Salem M. (2022). A Machine Learning Model for Torsion Strength of Externally Bonded FRP-reinforced Concrete Beams. Polymers.

[19] Amin M.N., Iqbal M., Khan K., Qadir M.G., Shalabi F.I., Jamal A. (2022). Ensemble Tree-based Approach Towards Flexural Strength Prediction of FRP Reinforced Concrete Beams. Polymers.

[20] Amin M.N., Iqbal M., Jamal A., Ullah S., Khan K., Abu-Arab A.M., Al-Ahmad Q.M.S., Khan S. (2022). GEP Tree-Based Prediction Model for Interfacial Bond Strength of Externally Bonded FRP Laminates on Grooves with Concrete Prism. Polymers.

[21] Liu Y., Pan F., Xiong F., Wei Y., Ruan Y., Ding B., Yang K., Chen Y. (2023). Ultrafast Shape-Reconfigurable Chiral Mechanical Metamaterial based on Prestressed Bistable Shells. Adv. Funct. Mater..

[22] Hyer M.W., White S.R. (2009). Stress Analysis of Fiber-reinforced Composite Materials.

[23] Hoskin A. (2018). Blossoming of Coiled Deployable Booms. Ph.D. Thesis.

[24] Viquerat A., Schenk M., Lappas V., Sanders B. Functional and Qualification Testing of the InflateSail Technology Demonstrator. Proceedings of the 2nd AIAA Spacecraft Structures Conference.

